# *Rickettsia africae* and Novel Rickettsial Strain in *Amblyomma* spp. Ticks, Nicaragua, 2013

**DOI:** 10.3201/eid2402.161901

**Published:** 2018-02

**Authors:** Helena Vogel, Janet Foley, Christine V. Fiorello

**Affiliations:** University of California, Davis, California, USA

**Keywords:** ticks, dogs, rickettsiae, *ompA*, Nicaragua, bacteria, *Rickettsia* *africae*, *Amblyomma* spp., vector-borne infections

## Abstract

We report molecular detection of *Rickettsia* *africae* in *Amblyomma* *ovale* ticks from Nicaragua and a novel rickettsial strain in an *A. triste* tick. Of 146 ticks from dogs, 16.4% were *Rickettsia* PCR positive*.* The presence of *Rickettsia* spp. in human-biting ticks in Nicaragua may pose a public health concern.

Obligately intracellular *Rickettsia* spp., typically transmitted by ticks, cause a multitude of mild to severe rickettsial diseases in humans and other animals. Novel *Rickettsia* species have been identified through molecular techniques (*1*). Rickettsiae in Central America have primarily been reported in ticks, dogs, and humans, with limited data on tick species and rickettsial prevalence in Nicaragua (*1*). In an earlier study, 87% of 77 dogs in the Bosawás Biosphere Reserve were seropositive for rickettsiae (*2*); the ticks in that study were collected from 40 of those dogs.

The Bosawás Reserve in remote northern Nicaragua, part of the second largest tropical rainforest in the Western Hemisphere, is inhabited by 2 rapidly growing populations of indigenous people: the Miskito and the Mayangna. These subsistence-based communities use dogs for hunting in the reserve. Increasing connectivity with outside areas, population growth, and interference of dogs with wildlife pose an increased risk for the emergence of zoonotic rickettsioses. We planned to expand information on zoonotic *Rickettsia* spp. in Nicaragua by surveying ticks from hunting dogs for diversity, number, and presence of rickettsiae.

We collected ticks in 2013 from villages at similar latitude and longitude measured by using global positioning system (GPS): Arang Dak (14.51583, −84.99944), Amak (14.06542, −85.142233), and Raiti (14.59464, −85.02772) (Table). Arang Dak is the smallest of the 3 villages and closest to the densest part of the rainforest; Raiti is the largest and most developed village of the 3 and is situated on a heavily traveled route through the reserve. We obtained owner consent before physical examination and sampling of ticks from dogs and stored ticks in 70% ethanol. In the laboratory, we identified ticks for sex, life stage, and species by using a key (*3*) and screened tick DNA for *Rickettsia* spp. by real-time PCR (*4*). *Rickettsia-*positive samples were further tested by conventional PCR targeting the outer membrane protein A gene (*ompA*) (*5*). We also amplified the *rpmB* and *17kDa* genes of the rickettsia in the *Amblyomma triste* ticks we recovered (*4*). We sequenced each amplicon by using the forward primer at University of California Davis Sequencing (Davis, CA, USA) and compared sequences to those in the GenBank database by using the BLAST algorithm (https://blast.ncbi.nlm.nih.gov).

**Table:** Number of dogs and ticks sampled, tick species, and prevalence of rickettsiae in ticks in 3 indigenous communities, northern Nicaragua

Category	Amak	Raiti	Arang Dak	Total
No. dogs sampled	11	10	19	40
No. ticks collected and tested	25	55	66	146
*A. ovale* (PCR-positive)	25 (4)	45 (10)	57 (4)	127 (18)
*A. sculptum* (PCR-positive)	0	4 (3)	8 (2)	12 (5)
*A. triste* (PCR-positive)	0	6 (1)	1 (0)	7 (1)
Prevalence of rickettsiae in ticks, % (95% CI)	16.0 (5.25–36.9)	25.5 (15.1–39.3)	9.09 (3.75–19.4)	16.4 (11.0–23.7)

Of 146 ticks from 40 dogs, 126 (86%) were *A. ovale*, 12 were *A. sculptum*, and 7 were *A. triste*. We detected rickettsial DNA in 24 (16.4%, 95% CI 11.0%–23.7%) of the 146 ticks: 18 *A. ovale*, 5 *A. sculptum*, and 1 *A. triste*. We deposited rickettsial sequences from these ticks into GenBank (accession no. KX530472, KX576685, and KX576686). 

By location, the PCR prevalence was 25.5% (95% CI 15.1%–39.3%) in Raiti, 16.0% (95% CI 5.25%–36.9%) in Amak, and 9.09% (95% CI 3.75%–19.4%) in Arang Dak. These differences were statistically significant (p = 0.05 by Fisher exact test). The finding of highest prevalence in the most populated community is consistent with peridomestic animals maintaining the infection, and the rainforest and remote wildlife not being significant sources.

For the 576-bp *ompA* sequence, all from *A. ovale* ticks were identical and were 99.6% homologous with sequences from GenBank identified as *R. africae*. *R. africae* has not been reported in *A. ovale* ticks or in North, Central, or South America. *R. africae* causes a mild rickettsiosis known as African tick-bite fever and was first described in a patient in the Western Hemisphere in 1998 (*1*). *R. africae* has been detected in *A. variegatum* ticks by using PCR and in humans in Guadeloupe by using serology (*6*) and more recently in *A. loculosum* ticks from New Caledonia (*7*). In Brazil, adult *A. ovale* ticks bite humans most frequently and are present from the borders of Mexico to those of Argentina (*8*)*.* *A. ovale* is a common human-biting tick in Central and South America and poses a public health concern.

Sequences of *ompA* in 2 of 5 PCR-positive *A. sculptum* matched 99.6% to *Candidatus* R. amblyommii in GenBank (*ompA* of the other samples did not amplify, likely because they were relatively weak on real-time PCR). *Candidatus* R. amblyommii is common among *Amblyomma* spp. ticks in the New World and was reported in *A. sculptum* ticks in Brazil (*9*). *Candidatus* R. amblyommii has unknown pathogenicity but has been implicated in rickettsiosis cases in humans (*9*).

The *ompA* amplicon from *A. triste* ticks matched *Rickettsia* sp. ARAGAOI; sequencing of the *rpmB* and *17kDa* genes was unsuccessful. This rickettsial species was originally described in marsupials in Brazil (*10*). Further monitoring of tick vectors in this remote area is needed to characterize local risk and detect possibly emerging vectorborne disease.
