# Correction: Vol. 24, No. 2

**DOI:** 10.3201/eid2406.C32406

**Published:** 2018-06

**Keywords:** errata, erratum, correction

*Amblyomma mixtum* ticks were misidentified as *A. sculptum* in *Rickettsia africae* and Novel Rickettsial Strain in *Amblyomma* spp. Ticks, Nicaragua, 2013 (H. Vogel et al.). The article has been corrected online (https://wwwnc.cdc.gov/eid/article/24/2/16-1901_article).

